# The Scope of Infirmary Surgery To-Day

**Published:** 1913-04-05

**Authors:** 


### INFIRMARY OPERATIONS.

### The Scope of Infirmary Surgery To-day.

The infirmary medical officer gets ample opportunities to perfect himself in surgical technique. It should be his endeavour to take the utmost advantage of these chances and to make himself proficient in both major and minor surgery. In a small institution, where he is practically single-handed, he must make it his duty to train one or two of the nursing staff to act as his assistants. This must be done systematically and thoroughly if such assistance is to be of real value to him. The principles of surgical asepsis must be thoroughly grounded into the nurses; they must be taught that it is nothing less than a crime to venture upon an operation, however trivial it may seem, without the rigorous ritual prescribed by modern surgical science. Constant teaching and example alone can bring perfection in this, but the trouble and time spent in inculcating these essential principles are amply repaid by the subsequent good results which are to be attained.

Nor must the surgeon think that because he does not possess the elaborate apparatus familiar to him in his student days that he cannot therefore undertake major operations. We know that it is a growing tendency on the part of some superintendents to make their operating theatres rival in sumptuousness and elaborateness the richly equipped theatres which some years ago were fashionable in general hospitals. Such men have surely failed to realise that the modern practice is to simplify, not to elaborate. The simpler the arrangements in the operating theatre the better. We know of a small infirmary which possesses a comparatively ill-equipped theatre—if the little room with its old-fashioned screw table can be dignified by that name—in which, nevertheless, some of the best surgery we have seen has been carried out by an infirmary surgeon whose greatest characteristic is his scrupulous insistence on asepsis. Such insistence does not, again, mean the command of elaborate disinfecting and sterilising apparatus; it means only a proper regard for cleanliness in everything that touches patient or operator. A liberal use of soap and water, aided by the use of sterilised gloves—an aid which every infirmary officer should insist upon having—can secure that perfection of asepsis without which every operation entails a considerable amount of risk. Gloves of thin rubber are nowadays so cheap that a plentiful supply of them should be secured. If bought in large quantities they can be kept ready for use in a locker or cabinet in the operating-room itself or in an adjacent instrument room if such exists.

The question of anæsthetics is one which the infirmary surgeon will sometimes find difficult, especially if he has little expert assistance. In spite of skilled assistance, the infirmary surgeon may have to turn to local or spinal anæsthesia for his choice. In such a case he should make a point of carefully studying the technique and frequently witnessing operations done under local anæsthesia by surgeons working at the general hospitals. New methods are constantly arising, and only by following the literature and making himself acquainted with what progress is done in this direction can he achieve success and avoid difficulty and trouble. For him especially local anæsthesia, combined with a general narcotic, is advisable in single-handed operations. This method robs local anæsthesia of its terrors—which we are too apt to underestimate, especially in old and feeble patients and in children—and gives him an added safeguard. Needless to say local anæsthesia has its dangers and disadvantages, against which the surgeon must guard.

Operating under difficulties, on patients whose vitality is often sapped through underfeeding and drink, the infirmary surgeon will find that his percentage of "clean cases" may not at first compare at all favourably with what he had to his credit in his hospital days as house surgeon. Attention to details, strict asepsis, and careful preparation of the patients, wherever such preparation is possible, will raise that percentage considerably. Yet, as a general rule, it must be admitted that the type of infirmary case compares unfavourably as regards stamina and vitality with the ordinary class of hospital cases. That is an unfortunate fact which the surgeon must bear in mind, and he must not be discouraged if his successes in major operations are not so excellent as he may at first have expected. The skilled nursing in the general hospitals is undoubtedly an added factor in this comparison, but it is a factor which the infirmary superintendent can make influential in his own institution by properly training his nursing staff in surgical work.

The class of surgery that comes to the infirmary officer is exceedingly varied, and he must be prepared to undertake every kind of operation, from the extraction of a tooth stump to a Cæsarean section. On his intrepidity and skill alone depends the choice of what he shall tackle, and it is therefore well if he keep himself fully abreast of the latest methods, and thoroughly familiarises himself with the best instruments. Much more than is nowadays done may be achieved in infirmary practice if the staff would take the trouble to become acquainted with the proper handling of the newer instruments for so-called diagnostic operations. We refer especially to brain puncture, œsophagoscopy, rectoscopy, and cystoscopy. It is true these instruments are expensive, and their technique is perhaps difficult to learn without previous practice under a good instructor. But a few demonstrations will enable the tyro to start practice on his own, and with care and discrimination he will soon learn how to perfect himself. The help that he can obtain from a successful use of these newer aids to diagnosis is often invaluable. Similarly, with regard to newer methods of cure. Re-section of a deviated septum is another simple operation in the majority of cases, and although these may be styled, with some show of justification, as primarily operations of convenience and not of necessity, there are numerous occasions when they give relief.

